# Animal Coronavirus Diseases: Parallels with COVID-19 in Humans

**DOI:** 10.3390/v13081507

**Published:** 2021-07-30

**Authors:** Chao-Nan Lin, Kuan Rong Chan, Eng Eong Ooi, Ming-Tang Chiou, Minh Hoang, Po-Ren Hsueh, Peck Toung Ooi

**Affiliations:** 1 Department of Veterinary Medicine, College of Veterinary Medicine, National Pingtung University of Science and Technology, Pingtung 91201, Taiwan; mtchiou@mail.npust.edu.tw; 2 Animal Disease Diagnostic Center, College of Veterinary Medicine, National Pingtung University of Science and Technology, Pingtung 91201, Taiwan; 3 Program in Emerging Infectious Diseases, Duke-NUS Graduate Medical School, Singapore 169857, Singapore; kuanrong.chan@duke-nus.edu.sg (K.R.C.); engeong.ooi@duke-nus.edu.sg (E.E.O.); 4 Viral Research and Experimental Medicine Centre (ViREMiCS), SingHealth Duke-NUS Academic Medical Centre, Singapore 169856, Singapore; 5 Saw Swee Hock School of Public Health, National University of Singapore, Singapore 117549, Singapore; 6 Department of Anatomy and Histology, College of Veterinary Medicine, Vietnam National University of Agriculture, Hanoi 100000, Vietnam; kira2108.hua@gmail.com; 7 Departments of Laboratory Medicine and Internal Medicine, China Medical University Hospital, School of Medicine, China Medical University, Taichung 404332, Taiwan; 8 Departments of Laboratory Medicine and Internal Medicine, National Taiwan University Hospital, National Taiwan University College of Medicine, Taipei 10051, Taiwan; 9 Faculty of Veterinary Medicine, Universiti Putra Malaysia, UPM, Serdang 43400, Selangor, Malaysia

**Keywords:** animal coronavirus, COVID-19, DIVA, immunopathogenesis

## Abstract

Coronavirus disease 2019 (COVID-19), caused by severe acute respiratory syndrome coronavirus 2 (SARS-CoV-2), a novel coronavirus in humans, has expanded globally over the past year. COVID-19 remains an important subject of intensive research owing to its huge impact on economic and public health globally. Based on historical archives, the first coronavirus-related disease recorded was possibly animal-related, a case of feline infectious peritonitis described as early as 1912. Despite over a century of documented coronaviruses in animals, the global animal industry still suffers from outbreaks. Knowledge and experience handling animal coronaviruses provide a valuable tool to complement our understanding of the ongoing COVID-19 pandemic. In this review, we present an overview of coronaviruses, clinical signs, COVID-19 in animals, genome organization and recombination, immunopathogenesis, transmission, viral shedding, diagnosis, treatment, and prevention. By drawing parallels between COVID-19 in animals and humans, we provide perspectives on the pathophysiological mechanisms by which coronaviruses cause diseases in both animals and humans, providing a critical basis for the development of effective vaccines and therapeutics against these deadly viruses.

## 1. Overview of Coronaviruses

Coronaviruses are enveloped viruses with a large, capped, and polyadenylated RNA genome of approximately 24,500–31,800 nucleotides [1], belonging to the genus *Coronavirus*, family *Coronaviridae*, order *Nidovirales*. The genus *Coronavirus* can be subdivided into four clusters based on genetic and serologic properties (i.e., *Alphacoronavirus*, *Betacoronavirus*, *Gammacoronavirus,* and *Deltacoronavirus*) [1]. *Alphacoronavirus* includes transmissible gastroenteritis virus (TGEV) in swine, porcine respiratory coronavirus (PRCV), porcine epidemic diarrhea virus (PEDV), swine acute diarrhea syndrome-coronavirus (SADS-CoV), canine coronavirus (CCoV), feline coronavirus, ferret and mink coronaviruses, human coronavirus (HCoV) 229E, HCoV NL63, and bat coronaviruses (Bat CoVs). *Betacoronavirus* includes murine hepatitis virus (MHV), bovine coronavirus, equine coronavirus, canine respiratory coronavirus (CRCoV), HCoV OC43, HCoV HKU1, Human enteric CoV-4408 (HECoV-4408), porcine hemagglutinating encephalomyelitis virus, rat coronavirus, severe acute respiratory syndrome coronavirus (SARS-CoV), Middle East respiratory syndrome-related coronavirus (MERS-CoV), SARS-CoV-2, and Bat CoVs. *Gammacoronavirus* includes avian coronaviruses, such as avian infectious bronchitis virus (IBV), turkey coronavirus (TCoV), goose coronavirus, duck coronavirus, and Asian leopard cat coronavirus. Interestingly, beluga whale coronavirus belongs to *Gammacoronavirus*. Finally, *Deltacoronavirus* includes wigeon coronavirus, bulbul coronavirus, moorhen coronavirus, munia coronavirus, thrush coronavirus, and porcine deltacoronavirus (PDCoV) [2].

Tracing the history of coronaviruses, the first coronavirus-related disease recorded may have been a case of feline infectious peritonitis (FIP), discovered as early as 1912 [3]. Diseases associated with avian IBV, TGEV, and MHV were well-known before 1950. However, the first human coronavirus was not identified until the late 1960s [4] and was responsible for causing the common cold. In late 2002, a novel human coronavirus, SARS-CoV, emerged in southern China. The disease spread rapidly, with over 8000 cases and about 800 deaths reported in 29 countries. The global SARS-CoV outbreak ended in July 2003 [5]. The global epidemic of SARS-CoV brought coronaviruses to the attention of public health officials and academic virologists. In 2012, a novel zoonotic coronavirus, MERS-CoV, was identified in humans. The disease was transferred to humans from infected dromedary camels. Approximately 35% of MERS-CoV-infected patients died from the disease, although there is no evidence for sustained human-to-human community transmission [6]. To date, only a few outbreaks have occurred in Saudi Arabia, United Arab Emirates, and Korea [6]. In late 2019, we witnessed a global pandemic of the newly discovered coronavirus disease 2019 (COVID-19) [7]. The causative agent is SARS-CoV-2, which is highly similar to SARS-CoV with a sequence identity of approximately 80% [7]. COVID-19 has spread to more than 200 countries worldwide, with over 183 million reported cases and 3.9 million cumulative deaths as of 4 July 2021 [8].

## 2. Clinical Signs

Animal coronavirus diseases involve multiple body systems, such as gastrointestinal, respiratory, and central nervous systems, with clinical symptoms varying from encephalomyelitis, hepatitis, and nephritis to peritonitis (Table 1). FIP was the first recorded coronavirus-related disease, observed as early as 1912 [3]. It is a common progressive, fatal disease in domestic and non-domestic felids caused by FCoV [9]. Most FCoV infections are associated with mild to subclinical enteric infections, and only 5–12% of seropositive cats develop FIP [10,11]. Ocular and/or neurological manifestations often occur in the dry or non-effusive form of FIP. However, in the wet or effusive form of FIP, progressive abdominal distention and pleural effusion are caused by the accumulation of a highly viscous and protein-rich fluid in the peritoneal and pleural cavity [9]. The wet form of FIP in cats usually results in death within weeks to months.

TGEV, PEDV, and PDCoV cause diarrheal diseases in piglets and sows. The main clinical signs are mild to severe watery diarrhea, vomiting, and anorexia. Mortality can be extremely high (up to 100%) in neonatal piglets, as disease severity and the age of infected pigs are negatively correlated [12,13]. Most recently, a highly pathogenic enteric coronavirus, SADS-CoV, emerged in Southern China in 2016 with high mortality in suckling piglets [14]. In addition, another porcine coronavirus known as porcine hemagglutinating encephalitis virus causes vomiting and wasting disease in piglets. However, the disease is relatively infrequent worldwide [15]. 

Among rodents, MHV can induce hepatitis as well as neurological diseases and enteritis depending on the strain [16]. BCoV infections are associated with three distinct clinical signs in cattle, including calf diarrhea, hemorrhagic diarrhea in adult cattle, and respiratory diseases [17]. In canines, gastroenteritis and respiratory symptoms usually arise from CCoV (*Alphacoronavirus*) and CRCoV (*Betacoronavirus*), respectively [1]. The clinical manifestations of avian infectious bronchitis depend on the genetic background, age, route of infection, nutritional factors, virulence, and environmental stresses, such as low temperature or bacterial pathogen coinfections. Clinical presentations of IBV include respiratory diseases (gasping, coughing, tracheal rales, sneezing, nasal discharge, wet eyes, and swollen sinuses) and permanent hypoplasia of the oviduct in young female chicks [18]. TCoV can induce various enteric disease syndromes [19]. The taxonomy of coronaviruses, hosts, and clinical presentation in farm animals, rodents, and humans are summarized in Table 1. 

## 3. COVID-19 in Animals

COVID-19 is a zoonotic disease believed to have originated in animals [7]. However, emerging studies have shown that the disease can also be transferred from infected humans to animals, such as domestic and nondomestic animals [20]. Bats are reservoirs for SARS-CoV [21] and other CoVs related to MERS-CoV and SARS-CoV [22]. Therefore, bats are the most likely potential reservoir with a possible intermediate transmission event in pangolins for SARS-CoV-2 [23]. Among companion animals, dogs and cats have also been reported to be permissive for COVID-19 infection [24,25]. Dogs can shed low amounts of SARS-CoV-2 from nasal and/or oral swabs without clinical symptoms. In contrast, cats are more susceptible to SARS-CoV-2 than dogs in clinical cases [24]. All reported cases have an owner with COVID-19 or live in an area with a high incidence of COVID-19 [20]. The first feline case was reported in mid-March 2020 in Belgium, when a cat showed mild clinical signs, such as a loss of appetite, diarrhea, vomiting, cough, and shallow breathing. These signs appeared a week after the cat owner self-quarantined at home due to COVID-19 [20]. Subsequently, consecutive cases of SARS-COV-2 in cats were reported in several countries [22]. Cats with COVID-19 are usually asymptomatic or have mild to moderate respiratory and gastrointestinal symptoms [24]. Therefore, despite evidence for the human-to-cat transmission of SARS-CoV-2, cat-to-cat transmission has been successful only under experimental inoculation, and cat-to-human transmission remains unclear [24]. 

In early April 2020, nondomestic felids, such as tigers and lions, infected with SARS-CoV-2 were reported [20]. The zoo animals appeared to have mild respiratory symptoms and gradually recovered after receiving supportive treatment [20]. In addition, respiratory disease and increased mortality occurred in mink farms in the Netherlands [26]. Notably, several human cases of COVID-19 have been identified in Denmark with SARS-CoV-2 variants believed to have originated from farmed minks [27]. Previous studies have suggested that other farm animals, such as pigs, chickens, and ducks, have low susceptibility to COVID-19 [24]. However, ferrets and cats are highly susceptible to SARS-CoV-2 under experimental inoculation [24,28,29,30]. To date, there is evidence for SARS-CoV-2 transmission from humans to various animal species within the families *Caninae*, *Felinae*, and *Mustelidae* [20]. Therefore, it is necessary to implement preventive measures, including the use of personal protective equipment by veterinarians and related-animal workers, and to list these workers as priority groups for vaccination against COVID-19 around the world as the risk of indirect infection remains high.

## 4. Genome Organization and Recombination

Positive-sense single-stranded RNA viruses have approximately 10^−2^ to 10^−5^ nucleotide substitutions per site per year [31]. Similar to other RNA viruses, coronaviruses show a high mutation frequency due to high rates of RNA polymerase errors [32]. The genome organization of coronaviruses contains non-structural proteins (NSPs), four structural proteins (spike (S), envelope (E), membrane (M), and nucleocapsid (N) and several accessory genes [1]. Based on structural studies, bioinformatic analyses, and biochemical experiments, SARS-CoV-2 appears to be optimized for binding to ACE2, possessing a receptor-binding domain (RBD) of the spike protein, which has a high affinity to ACE2. This is in contrast to animal coronaviruses, which bind to human ACE2 with lower affinity [33]. Another notable feature that distinguishes SARS-CoV-2 from the other animal coronaviruses is the presence of a polybasic cleavage site (RRAR), which allows more effective cleavage by furin and other proteases, thereby increasing viral infectivity and influencing host tropism [33].

Non-structural genes encoding polyproteins 1ab are subdivided into approximately 16 NSPs involved in proteolytic processing, genome replication, and subgenomic mRNA synthesis [1]. The S protein of coronavirus plays key roles in binding to host cell surface receptors for viral entry and eliciting neutralizing antibodies that contribute to protective immunity [1]. Unsurprisingly, several SARS-CoV-2 variants have been reported, such as Alpha, Beta, Gamma, and Delta variants, with mutations in the S gene [34]. Similar characteristics have been reported in a porcine coronavirus, PEDV, which was neglected until an outbreak of a new variant in China in 2010. To date, several PEDV variants have been noted in the swine industry worldwide [35,36]. A mutation in the S gene is associated with the FCoV biotype [37,38,39]. Virulent FCoV can efficiently replicate in monocytes/macrophages, which can subsequently spread and circulate within the body, whereas avirulent FCoV replicates only in the gut epithelium [38]. In addition to non-structural and structural proteins, the genomes encode several accessory proteins in FCoV [4,40,41,42,43,44,45,46,47,48,49]. Accessory genes, which encode proteins that are non-structural and not essential for viral replication in vitro, although they are believed to play roles in host immune response [40]. The specific roles of the accessory proteins of different coronaviruses are still poorly understood and warrant further investigation.

A unique feature of coronaviruses is the high frequency of genome recombination events during the evolution of the lineage [32]. Recombination among coronaviruses is thought to contribute to the emergence of new pathotypes, such as SARS-CoV [50,51], HCoV NL63 [52], HCoV HKU1 [53], IBV [54], CRCoV [55], TGEV [56,57], and FCoV [48,58]. FCoVs are further classified into two serotypes [59]. Type I viruses are believed to be the ancestral FCoV, and type II FCoVs may be derived from an individual and double recombination event between type I FCoV and CCoV [48,58]. The progeny viruses contain one-third of the CCoV genome [48]. Both types of FCoV can cause FIP and enteric infection [59]. The recombination of coronaviruses is significantly correlated with fatal FIP and increased transmission between cats in clinical settings [49,60]. In addition, the common replication signaling elements among *Betacoronavirus* suggest a high potential for recombination within group members [61]. Cross-species transmission is thus worthy of further investigation. For example, a *Betacoronavirus* HECoV4408 isolated from a child with diarrhea is thought to have originated from the bovine species [62]. Additionally, avian deltacoronavirus shows avian-to-swine transmission, as proven by molecular analyses [2]. Therefore, continuous and intensive surveillance of SARS-CoV is needed, especially in areas with MERS-CoV cases.

## 5. Immunopathogenesis

HCoV and avirulent FCoV, observed prior to the SARS-CoV outbreak, are non-pathogenic and induce mild inflammation. In contrast, SARS-CoV, SARS-CoV-2, and virulent FCoV are highly pathogenic coronaviruses in humans and cats, respectively. They cause diseases with similar immunopathogenic features; both are characterized by an intense inflammatory/cytokine storm that compromises normal physiological function and contributes to progressive, debilitating manifestations, such as fever and systemic disease [63,64,65]. Genetic factors associated with FIP have been identified [66,67,68]. Experimental FIP is a disease model for studies of coronavirus-related immunopathogenesis [69]. It can be classified into five groups based on the survival period, including rapid, intermediate, and delayed progression and prolonged or long-term survival. All cats present characteristic signs of acute viral infection within 7 days post-infection, in which neutralizing antibodies appeared and increased with identical kinetics in survivors and non-survivors. However, stronger cell-mediated immunity (CMI) was found in survivors than in non-survivals. These findings implied that the humoral response against virulent FCoV is insufficient to confer protection and that CMI is critical for controlling the infection and FCoV clearance [69]. Indeed, similar findings have been reported in patients with severe and moderate COVID-19 [70]. The early induction of strong T-cell responses is associated with an asymptomatic presentation or mild symptoms, whereas strong antibody titers are more closely linked to severe COVID-19 [71]. Moreover, the COVID-19 RNA vaccine efficacy is correlated with early T-cell responses rather than receptor-blocking antibodies, reinforcing the importance of CMI for protection against COVID-19 [72].

Similar to FIP caused by virulent FCoV, severe COVID-19 is associated with the substantial suppression of natural killer (NK) cells and regulatory T (Treg) cells, as reflected by lower cell counts and reduced NK cell functionality [70,73,74]. As Treg cells can counter-regulate immune responses and control undesired immune responses, the reduced quantity of Treg cells may contribute to the inflammatory/cytokine storm, attributed to the dysregulation of the host immune system [75]. The over-production of tumor necrosis factor-alpha (TNF-alpha) is associated with a poor prognosis in patients with SARS-CoV and MERS-CoV infections [76]. Interestingly, similar immunological features have also been reported in FIP cases, in which TNF-alpha production contributed to the aggravation of FIP [77,78]. Increased TNF-alpha production is associated with viral replication in virulent FCoV-infected macrophages and under antibody-dependent enhancement (ADE) [78], supporting the link between the viral load and TNF-alpha production. Increased TNF-alpha levels can then act on macrophages and promote FCoV receptor expression [78] and are responsible for the induction of apoptosis in CD8^+^ T cells [77].

Antibodies that bind to S proteins might contribute to immunopathogenesis, especially if they are weakly neutralizing or present at sub-neutralizing levels. Antigen–antibody complexes that are deposited in the blood vessel walls and induce complement activation can lead to vasculitis and edema, contributing to the development of the effusive form of FIP [9]. The opsonized virion might also lead to enhanced infection in Fc-gamma receptor-bearing cells, including monocytes, macrophages, and dendritic cells, via Fc-gamma receptor-mediated uptake, a phenomenon known as ADE [79]. Increased uptake of virus–antibody complexes by Fc-gamma receptor-bearing cells can consequently promote virus propagation, thereby increasing disease severity in infected individuals or animals [80]. ADE is not unique to FCoV and has been widely documented in viral taxa, such as dengue viruses, Zika virus, Ebola virus, and human immunodeficiency virus (HIV) [79].

The evasive mechanisms associated with ADE have yet to be thoroughly investigated. However, recent studies have suggested that interactions between antibody-opsonized virions and other surface receptors, such as leukocyte Ig-like receptor-B1 suppress antiviral responses [81]. In addition, virus–antibody complexes may induce the early expression of host dependency factors involved in various pathways, such as RNA splicing, mitochondrial respiratory chain complexes, and vesicle trafficking, thereby promoting viral replication [82]. Importantly, Lee et al. described two plausible mechanisms underlying ADE in COVID-19: (1) ADE via enhanced infection and (2) ADE via enhanced immune activation [83]. Although the effect is yet to be established, anti-SARS-CoV-2 antibodies could exacerbate COVID-19 [83]. However, ADE has not been reported after more than 1000 million vaccinations against COVID-19 in humans, likely due to the ability of the vaccines to induce sufficient neutralizing antibodies. However, due to the high SARS-CoV-2 mutation rate, the antibody affinity and neutralizing antibody levels against SARS-CoV-2 might change over time. Therefore, given that ADE can exacerbate the pathogenesis of FCoV infection, its contribution to COVID-19 outcomes still needs to be closely monitored.

## 6. Transmission and Viral Shedding

**Figure 1:**
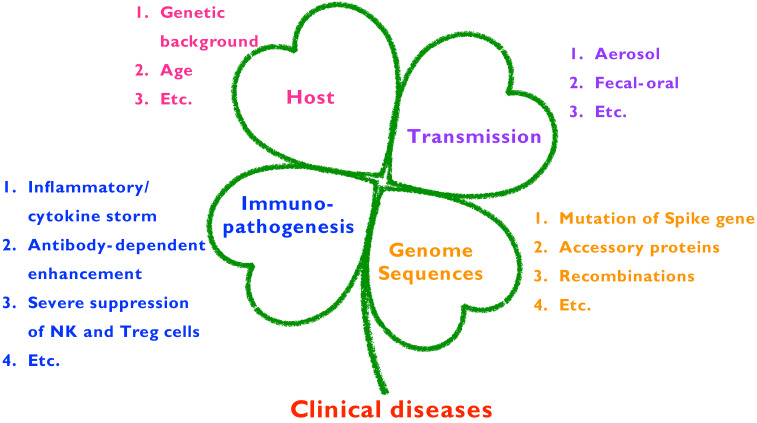
General summary of hosts, genome sequences, immunopathogenesis, and transmission features of animal coronaviruses that lead to clinical disease.

Enterotropic coronaviruses, such as TGEV, PEDV, PDCoV, SADS-CoV, CCoV, BCoV, TCoV, and enterotropic MHV, can be transmitted by direct fecal-oral or indirect routes. Several coronaviruses are spread by aerosol droplets and the ingestion of contaminated food or water. Therefore, research on the viral shedding period is important. In a natural PEDV outbreak, high levels of PEDV shed by feces can be seen for a few days post-infection, and the viral titer gradually decreases after one week. However, intermittent viral shedding can be detected up to 2 months post-infection [84]. In a 7-year longitudinal monitoring study, under a multi-cat environment, cats that recovered after transient infection were subsequently re-infected with either the same or a different FCoV strain [85]. Adult cats shed FCoV in their feces intermittently at least once during the year, whereas the median age at which FCoV was first detected in kitten feces was 67 days old (range, 33 to 78 days) [86]. A recent study has indicated that FCoV antibody titers are correlated with the likelihood and frequency of FCoV shedding and fecal viral load [87]. Chronic shedders have higher antibody titers and shed more FCoV in their feces. In general, cats infected with FCoV usually begin fecal viral shedding after one week, followed by three possible outcomes in which cats (i) become chronic FCoV carriers, persistently shedding the virus for various durations or lifelong; (ii) eliminate the infection and stop shedding FCoV but can become re-infected; or (iii) continuously or intermittently shed FCoV (observed in the majority of cats) [88]. This information highlights the importance of COVID-19 monitoring; however, examinations of viral shedding by rectal swab might not be feasible in humans. Host information, genome sequences, immunopathogenesis, and transmission features of animal coronaviruses (PEDV and FCoV) and SARS-CoV-2 are presented in Figure 1.

## 7. Diagnosis

Serological and molecular tests are often utilized for the detection of specific antibodies and antigens in veterinary and human infectious diseases. However, several issues are of concern with respect to the clinical diagnosis of coronaviruses. 

**Figure 2:**
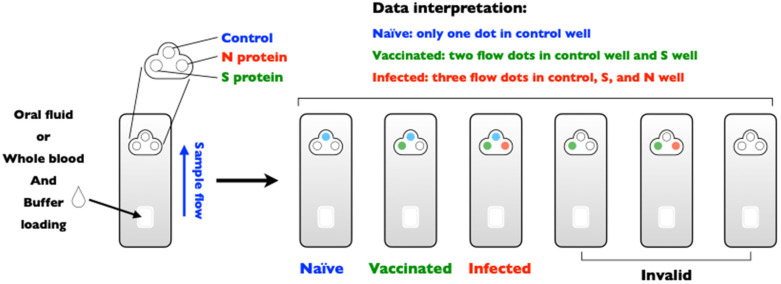
DIVA strategy for SARS-CoV-2 serological testing. The usage of a DIVA assay kit to differential naïve, vaccinated, and/or infected, with invalid results included.

(i) Is the presence of the virus and antibodies an indicator of disease status? The answer is sometimes not straightforward. For instance, when PEDV infects naïve pigs, especially young pigs, the disease manifestation can be very severe. However, during the recovery phase after PEDV infection, the lack of long-term persistent infection would indicate that the risk of virus transmission is reduced [84]. In contrast, FCoV is a ubiquitous virus in the feline population with a seroprevalence of 0–87% and much higher rates in multi-cat environments than in single cat households and in stray cats [88]. As the infection rates of FCoV are high in cats, the presence of the virus may not predict the development of FIP. Indeed, several reports have indicated that 5–12% of seropositive cats eventually develop FIP [10,11], depending on several factors, including host-related factors [66,67,68], environment-related factors [88], and virus-related factors [37,38,44,60]. Therefore, early diagnosis of FIP remains one of the biggest challenges for veterinarians. Although quantitative reverse-transcription polymerase chain reaction (qRT-PCR) is a powerful tool for molecular diagnosis, it cannot distinguish virulent from avirulent FCoVs [68]. Taken together, current data suggest that the presence of PEDV may be linked to disease status, whereas this may not be the case for FCoV infection. COVID-19 is an ongoing global pandemic; however, it might become seasonal with global vaccination against SARS-CoV-2 [89]. At present, methods for the detection of novel virulent SARS-CoV-2 variants are needed. 

(ii) Is differentiating infected from vaccinated animals (DIVA) possible? The principle of DIVA is to test the possibility of serological surveillance for the presence of wild-type infection. The DIVA strategy is available for some important transboundary animal diseases, such as foot-and-mouth disease, classical swine fever, and avian influenza [90]. However, this strategy does not appear to be effective for animal coronavirus vaccination, which may be a limitation of immunity passports for SARS-CoV-2 [91]. Nonetheless, the DIVA strategy for COVID-19 might still be a useful means to measure the presence of breakthrough infections, even in individuals who have previously been vaccinated. Figure 2 describes the DIVA strategy for the detection of anti-N antibodies, which are only elicited in individuals exposed to the whole SARS-CoV-2 virus, whereas anti-S antibodies can be detected in individuals who were previously vaccinated [92] (inactivated, mRNA, replication-incompetent vectors, or recombinant S protein vaccines) or exposed to SARS-CoV-2 infection. Importantly, DIVA can be applied for antibody detection in oral fluids, which is convenient and feasible for specimen collection and can show high sensitivity and specificity if properly optimized [93]. 

(iii) Can the specificity and sensitivity of diagnostic tools, especially novel diagnostic methods and strategies, be improved? At present, viral nucleic acid detection by molecular tests remains the gold standard for COVID-19 [94]. However, to survey a larger population, a more cost-effective pooling strategy will be essential, although the sensitivity for pooled samples remains to be evaluated. Nonetheless, pooled testing systems have been developed for large-scale epidemiological surveys, as well as for active surveillance and monitoring for PEDV control [95]. Based on lessons from animal coronaviruses, a pooled testing system could provide a road map for COVID-19 diagnosis.

## 8. Treatment

Supportive care is usually required in animal coronavirus cases. For FIP, treatment is focused on reducing the inflammatory and hyperimmune response, with several anti-FCoV agents and immunosuppressants also considered. Drugs that target FCoVs include carbohydrate-binding agents (*Galanthus nivalis* agglutinin) [96,97,98], HIV protease inhibitors (nelfinavir) [96], interferons [99,100,101,102,103,104,105], nucleoside analogues (ribavirin and GS-441524) [106,107], anti-malaria drugs (chloroquine) [108], anti-fungal drugs (itraconazole) [109], immunosuppressants (cyclosporin A) [110,111], 3C-like proteases (GC376) [112,113], and peptides of heptad repeats of S protein of FCoV [114]. However, side effects should be considered, as many of these drugs have not been systematically tested in animals. For example, ribavirin is toxic in cats [99,115]. As mentioned above, TNF-alpha is a critical factor for FIP development in cats [77,78], and TNF-alpha inhibitors or TNF-alpha antibodies show clinical efficacy in the treatment of FIP [116,117].

**Figure 3:**
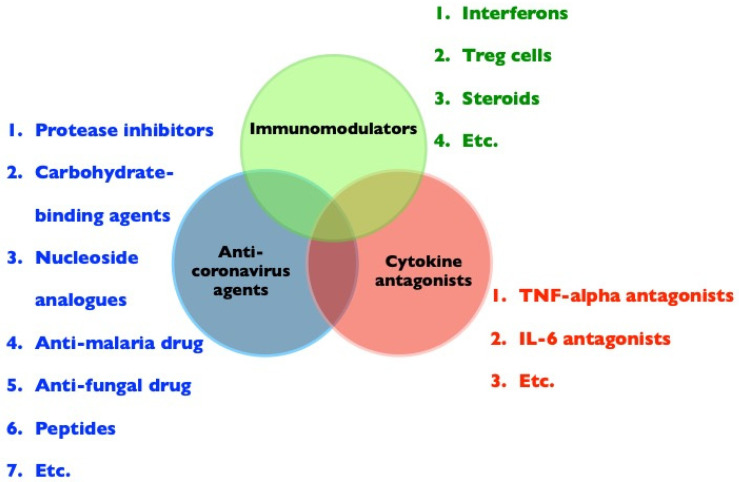
Potential treatment approaches for immunopathogenic diseases, such as FIP or COVID-19, including anti-coronavirus agents, cytokine antagonists, and immunomodulators.

The immunopathogenesis of FIP and COVID-19 are similar, and disease treatments aimed at the inhibition of coronavirus replication alone will not be sufficient. Indeed, the molecular pathways modulated by viruses and disease severity are temporally distinct [118]. Possible treatment strategies for immunopathogenic diseases caused by FCoV or SARS-CoV-2 are presented in Figure 3. Successful treatment approaches may include (i) antiviral agents, not limited to new antiviral agents, but including available drugs identified by screening [119]; (ii) cytokine antagonists, such as TNF-alpha inhibitors, TNF-alpha antibodies, or TNF-alpha receptor blockers to inhibit TNF-alpha-induced neutrophilia [120], apoptosis of T-lymphocytes [77], and viral receptor expression in target cells [78], and IL-6 antagonists to induce an increase in Treg cells [121]; and (iii) immunomodulators, i.e., interferons to induce host antiviral responses and immunomodulatory activity, which are widely used to treat human hepatitis [122]. Treg cells are significantly suppressed in both cats with FIP and patients with COVID-19 [70,73,74]. Therefore, Treg cells are potential immunotherapeutic agents due to their ability to counteract inflammatory responses [75]. In addition, prednisone or dexamethasone at immunosuppressive doses is the treatment of choice; however, such treatments are not curative and may only slow disease progression [88]. Therapeutic strategies that are focused on reducing both viral loads and inflammatory responses are most promising for pathologies associated with FIP and COVID-19.

## 9. Prevention

High-level biosecurity measures and vaccines are still the best strategies to prevent coronavirus diseases in animals and humans. Unfortunately, these diseases continue to spread worldwide due to the convenience of global trade [35,123]. For example, PEDV originated in Europe in the late 1970s [35] and became a problematic disease in China in 2010 [12], which subsequently spread to the United States (US) in 2013 [124]. Finally, the US-related PEDV strain expanded worldwide, reaching Taiwan [125], Japan [126], South Korea [127], Vietnam [128], Canada [13], and Mexico [35] and re-emerged in European countries in 2014 [84,129]. Therefore, high levels of biosecurity, such as changing protective equipment, washing exposed skin, or taking a shower, are recommended in pig farms and appear to be effective for reducing the risk of PEDV transmission [130]. In addition, strict quarantine is necessary to block the virus from entry into a country. Among highly devastating diseases, African swine fever (ASF), which is a highly contagious disease with clinical symptoms of hemorrhagic fever and leads to almost 100% mortality in domestic pigs [131], an extremely high level of biosecurity includes swab sampling from all areas and surfaces and testing by ASF qPCR assays [132]. A previous study has shown that FCoV can survive for 7 weeks in a dry environment [88]; accordingly, proper cleaning and disinfection are essential for the prevention and management of infections.

A recent review has shown that the neutralization levels of each type of vaccine (seven current vaccines) are highly predictive of immune protection [133]. As SARS-CoV-2 accumulates mutations [34], the efficacy of current vaccines might decrease. This may represent a leaky vaccine situation, and although an imperfect vaccine reduces pathogen virulence and disease severity, it does not completely protect against infection and transmission [134]. Therefore, it is likely that current COVID-19 vaccines may attenuate SARS-CoV-2 replication without completely eliminating the virus. A similar phenomenon has been reported in porcine circovirus type 2 (PCV2) and Marek disease virus in pigs [135] and chickens [136], respectively. PCV2 vaccination is one of the biggest success stories in veterinary medicine, with significant improvements in the average daily weight gain and mortality rate since vaccination worldwide [137]. In this case, the control of PCV2 is still effective, although the virus may continue to mutate and not completely eliminated from pigs [138,139].

## 10. Conclusions

Animal coronavirus diseases are emerging or reemerging worldwide. Veterinarians and animal coronavirus researchers are gaining experience and a better understanding of prevention and control strategies. Therapeutic strategies aimed at reducing viral loads and attenuating inflammatory/cytokine storms are the most promising against both FIP as well as COVID-19. Although COVID-19 was initially transmitted from animals to humans, we still cannot discount the potential for transfer from infected animals to humans and vice versa. Therefore, high-level biosecurity (personal protective equipment, physical distancing, and good hygiene), effective vaccines, and herd immunity are still the best strategies to prevent and control the spread of COVID-19. Finally, due to the similar features and characteristics of animal and human coronavirus, detailed insights from animal coronaviruses, especially porcine and feline coronaviruses, might provide a basis for understanding COVID-19.

## Figures and Tables

**Table 1:** Coronavirus taxonomy, hosts, and clinical presentation in farm animals, rodents, bats, and humans.

Subgenus	Virus	Host	Clinical Presentation			
			Respiratory	Enteric	Hepatitis	Neurologic
Alpha	Feline coronavirus (FCoV)	Cat	**√** ^1^	**√**	**√**	**√**
	Transmissible gastroenteritis virus (TGEV)	Pig	^2^	**√**		
	Porcine respiratory coronavirus (PRCV)	Pig	**√**			
	Canine coronavirus (CCoV)	Dog		**√**		
	Human coronavirus229E (HCoV-229E)	Human	**√**			
	Human coronavirus NL63 (HCoV-NL63)	Human	**√**			
	Porcine epidemic diarrhea virus (PEDV)	Pig		**√**		
	Swine acute diarrhea syndrome-coronavirus (SADS-CoV)	Pig		**√**		
	Bat coronaviruses (Bat CoV)	Bat				
Beta	Human coronavirus OC43 (HCoV-OC43)	Human	**√**			
	Human coronavirus HKU-1 (HCoV-HKU1)	Human	**√**			
	Human enteric coronavirus-4408 (HECoV-4408)	Human		**√**		
	Bovine coronavirus (BCoV)	Cow		**√**		
	Canine respiratory coronavirus (CRCoV)	Dog	**√**			
	Equine coronavirus (ECoV)	Horse	**√**			**√**
	Porcine hemagglutinating encephalomyelitis virus (PHEV)	Pig	**√**			**√**
	murine hepatitis virus (MHV)	Murine	**√**	**√**	**√**	**√**
	Middle East respiratory syndrome-related coronavirus (MERS-CoV)	Camel	**√**	**√**		
	Severe acute respiratory syndrome coronavirus (SARS-CoV)	Bat	**√**	**√**		
	Severe acute respiratory syndrome coronavirus 2 (SARS-CoV-2)	Bat	**√**	**√**		
	Bat coronaviruses (Bat CoV)	Bat				
Gamma	Infectious bronchitis virus (IBV)	Avian	**√**	**√**	**√**	
	Turkey coronavirus (TCoV)	Turkey		**√**		
Delta	Porcine deltacoronavirus (PDCoV)	Pig	**√**			

^1^ Common clinical manifestation. ^2^ Empty cells indicate few clinical manifestations or asymptomatic.

## Data Availability

Not applicable.
